# Red flags of poor prognosis in pediatric cases of COVID-19: the first 6610 hospitalized children in Iran

**DOI:** 10.1186/s12887-021-03030-2

**Published:** 2021-12-10

**Authors:** Sedigheh Madani, Sarvenaz Shahin, Moein Yoosefi, Naser Ahmadi, Erfan Ghasemi, Sogol Koolaji, Esmaeil Mohammadi, Sahar Mohammadi Fateh, Amirali Hajebi, Ameneh Kazemi, Erfan Pakatchian, Negar Rezaei, Hamidreza Jamshidi, Bagher Larijani, Farshad Farzadfar

**Affiliations:** 1 Non-Communicable Diseases Research Center, Endocrinology and Metabolism Population Sciences Institute, Tehran University of Medical Sciences, Tehran, Iran; 2 Endocrinology and Metabolism Research Center, Endocrinology and Metabolism Clinical Sciences Institute, Tehran University of Medical Sciences, Tehran, Iran; 3 Research Institute for Endocrine Sciences, School of Medicine, Department of Pharmacology, Shahid Beheshti University of Medical Sciences, Tehran, Iran

**Keywords:** COVID-19, Children, Iran, Epidemiology, Mortality

## Abstract

**Introduction:**

COVID-19 clinical course, effective therapeutic regimen, and poor prognosis risk factors in pediatric cases are still under investigation and no approved vaccinehas been introduced for them.

**Methods:**

This cross-sectional study evaluated different aspect of COVID-19 infection in hospitalized COVID-19 positive children (≺18 years oldwith laboratory confirmed COVID-19 infection, using the national COVID-19 registry for all admitted COVID-19 positive cases from February 19 until November 13,2020, in Iran.

**Results:**

We evaluated 6610 hospitalized children. Fifty-four percent (3268) were male and one third of them were infants younger than 1 year. Mortality rate in total hospitalized children was 5.3% and in children with underlying co-morbidities (14.4%) was significantly higher (OR: 3.6 [2.7-4.7]). Chronic kidney disease (OR: 3.42 [1.75-6.67]), Cardiovascular diseases (OR: 3.2 [2.09-5.11]), chronic pulmonary diseases (OR: 3.21 [1.59-6.47]), and diabetes mellitus (OR: 2.5 [1.38-4.55]), resulted in higher mortality rates in hospitalized COVID-19 children. Fever (41%), cough (36%), and dyspnea (27%) were the most frequent symptoms in hospitalized children and dyspnea was associated with near three times higher mortality rate among children with COVID-19 infection (OR: 2.65 [2.13-3.29]).

**Conclusion:**

Iran has relatively high COVID-19 mortality in hospitalized children. Pediatricians should consider children presenting with dyspnea, infants≺ 1 year and children with underlying co-morbidities, as high-risk groups for hospitalization, ICU admission, and death.

**Supplementary Information:**

The online version contains supplementary material available at 10.1186/s12887-021-03030-2.

## Introduction

Since Covid-19 outbreak in China, Iran was the first country in the Middle East that reported death due to COVID-19.According to COVID-19 Worldwide Dashboard - WHO Live World Statistics, Iran have had 2,640,670 confirmed cases and 74,524 deaths due to COVID-19 as of May 9, 2021 [1].

Although epidemiological characteristics, laboratory parameters, clinical course, risk factors for poor prognosis, and efficacy and safety of vaccination have been discussed in adults [2–8], infected children’s clinical course, effective therapeutic regimen, and poor prognosis’ risk factors are still under investigation.

Most of the pediatric cases have been shown to be mild or asymptomatic [9, 10], but there are matters that underline the importance of these patients: first, preliminary pediatric studies focus on the potential risk of COVID-19 transmission by pediatric carriers [11, 12], Second, more critical pediatric pulmonary manifestations have been observed specially in the case of the new variants of the virus [13, 14], third, sub-acute and chronic considerable complication of COVID-19 infection in children despite asymptomatic or pauci-symptomatic infection [15–17], and forth, growing cases of Multisystem Inflammatory Syndrome (MIS) of children have been observed [18–20], and have made this age group more important.

To the best of our knowledge, reliable evidence-based information about pediatric COVID patients has not been well studied to enable clinicians to plan accordingly. To the last version of world health organization interim guidance until November 10, 2020 [21], supportive care is the standard care for the infection. The effect of pharmacological treatments was under investigation but there are still some controversies. Also most available data were from developed countries and may not applicable in other countries [21, 22].

Present cross sectional study has summarized details of definite clinical outcome and clinical risk factors of mortality in all hospitalized Iranian children with positive results for COVID-19 Reverse Transcription Polymerase Chain Reaction (RT-PCR) up to November 13. We wish to highlight the red flag symptoms and characteristics in hospitalized children after justifying for age distribution and underlying co-morbidities.

## Methods

### Data collection and outcomes

Iran COVID-19 national registry collected all laboratory confirmed and probable acute COVID-19 cases throughout the country from February 19, 2020 (when the first cases were detected), until present. Physicians completed electronic questioners for Iranian electronic registry of COVID-19. We used the last output of this registry to design this cross sectional study for evaluating hospitalized children with acute infection, from February 19, 2020 until November 13, 2020.

This study data only has included hospitalized children (≤18 years old) with laboratory confirmed COVID-19 infection according to the World Health Organization (WHO) interim guidance [21, 22]. Children hospitalized due to MIS were not included. Demographic, epidemiologic, underlying co-morbidities, clinical symptoms, prescribed medications, ICU admission, ventilation requirement (except ventilation support during CPR process), and mortality data were extracted from electronic registry by two statistic specialist then clarified by a text mining specialist. Text mining codes were written for categorizing underlying co-morbidities by using Pythone™ (3.7) [23]. Finally, a pediatrician reviewed extracted data to specify the age, symptoms, co-morbidities, and laboratory data. Main outcomes were defined as ICU admission and death due to COVID-19 infection.

### Statistical analysis

STATA version 20 and R software (version 3.6.3) were used for statistical analysis and illustrating the figures. Mann-Whitney U Test was used to find the association between independent variables. We performed uni-variant logistic regression for crude odds ratio (OR) between mortality as dependent variable and age, gender, co-morbidities, and symptoms as independent variables. The models were built distinctly for all of the children, previously healthy children, and children with underlying co-morbidities. All underlying co-morbidities of hospitalized COVID-19 children were screened by our pediatrician and the relevant ones were selected for subgroup analysis. Multi-variant analysis was performed after adjusting for age, gender, and different types of co-morbidities to compute the OR of death in different underlying co-morbidities, symptoms, different age subgroups, and sex with confidence interval of 95%. *P*-value ≺0.05 was considered significant**.**

## Results

### Demographic and clinical features

We surveyed 6610 (2%) inpatient children (0-18 years) from total 328,541 patients with laboratory confirmed COVID-19 of Iran national COVID-19 registry. Boys (54.8%) were admitted more than girls (45.2%) in different age groups (Table 1).

**Table 1:** Descriptive statistics of hospitalized Iranian COVID-19 patients up to November 2020

Characteristic	Hospitalized PCR+ children without any co-morbidities (***N*** = 6090)	Hospitalized PCR+ children with co-morbidities (***N*** = 520)	***P***-value
Demographics [N(%)]			
Age (Mean[95% CI])	5.92 (5.78-6.06)	7.34 (6.84-7.85)	< 0.001
< 1 year	1161 (19.1%)	78 (15.0%)	< 0.001
1-5 year	2108 (34.6%)	144 (27.7%)	
6-12 year	1473 (24.2%)	144 (27.7%)	
13-17 year	1348 (22.1%)	154 (29.6%)	
Male sex	3327 (54.6%)	301 (57.9%)	0.15
Signs and Symptoms [N(%)]			
No symptoms	871 (14.3%)	74 (14.2%)	0.96
Fever	2592 (42.6%)	170 (32.7%)	< 0.001
Cough	2175 (35.7%)	206 (39.6%)	0.07
Headache	444 (7.3%)	47 (9.0%)	0.14
Sore throat	519 (8.5%)	44 (8.5%)	0.96
Myalgia	750 (12.3%)	80 (15.4%)	0.04
Dyspnea	1618 (26.6%)	221 (42.5%)	< 0.001
Diarrhea	715 (11.7%)	46 (8.8%)	0.04
Outcomes [N(%)]			
ICU admission	750 (12.3%)	108 (20.8%)	< 0.001
Ventilation support	452 (7.4%)	54 (10.4%)	0.01
Death	275 (4.5%)	77 (14.8%)	< 0.001

**Fig. 1:**
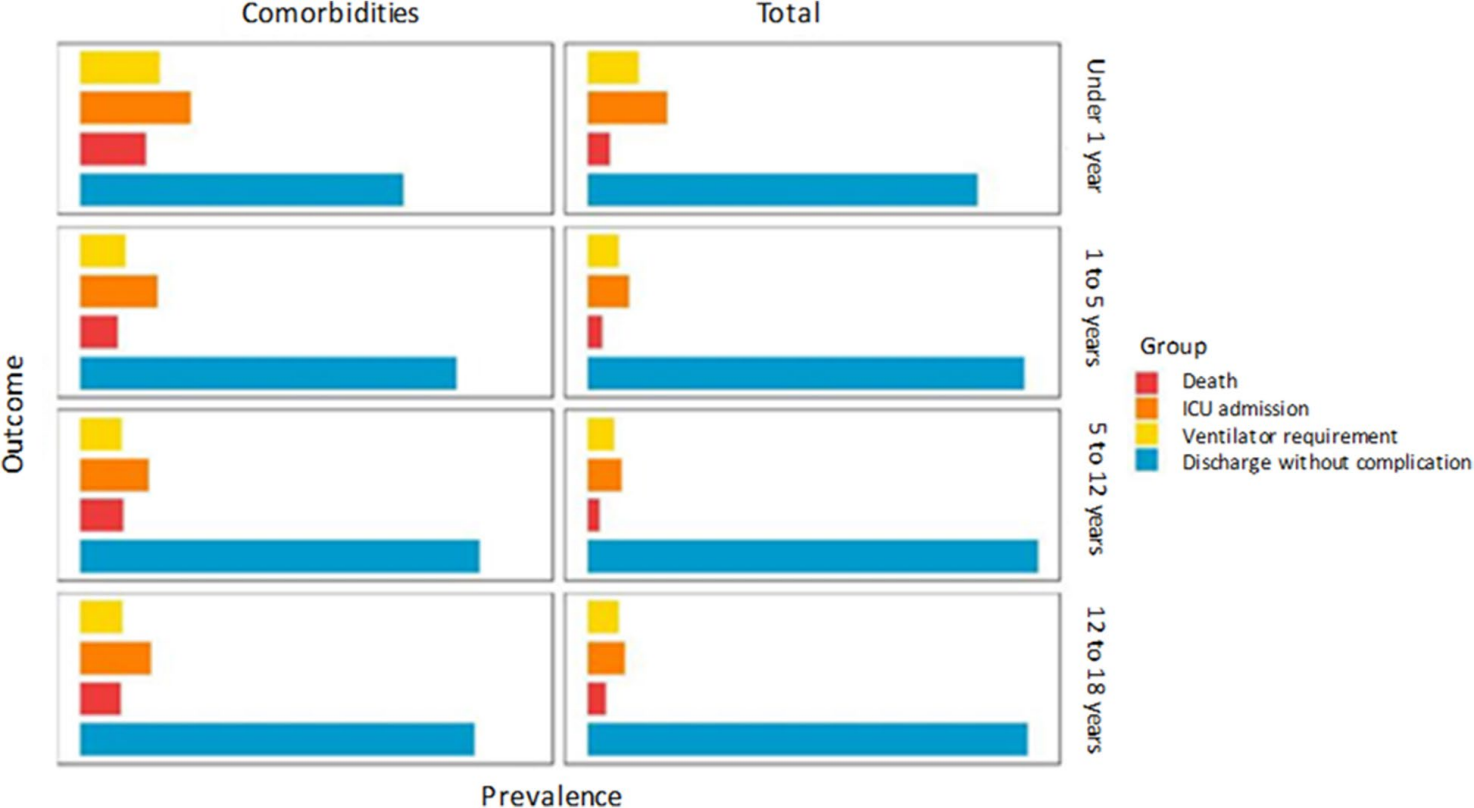
COVID-19 infection outcomes in Iranian hospitalized children in each age subgroup, considering previous health status of the children

More than half of the hospitalized children (53.7%) were younger than 5 years old. ICU admission and mortality rate in all hospitalized children were 13% (858/ 6610) and 5.3% (352/6610) respectively. Totally 7.6% (506/ 6610) of inpatient pediatric cases had life threatening respiratory failure and needed mechanical ventilation (Fig. 1 and Table 1).

**Fig. 2:**
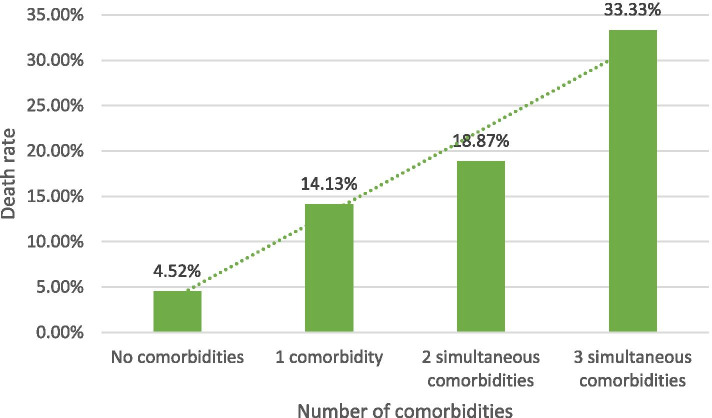
Rate of death in hospitalized COVID-19 children

Five hundred and nineteen co-morbidity was reported and consisted of cardiovascular diseases (2.8%), diabetes mellitus (1.6%), malignancies (1.5%), renal disorders (1%), chronic pulmonary disease (1%), liver disorders (0.5%), and immune deficiency disorders (0.6%) (Table 2). One, two, and three simultaneous co-morbidities were reported in 460, 53, and 6 of children respectively. Mortality rate in children with underlying co-morbidities was 14.8% (77 patients) and increased in multiple simultaneous co-morbidities (Fig. 2).

**Table 2:** Number (%) of patients and number (%) of deaths considering comorbidities in hospitalized Iranian COVID-19 patients divided by age groups

Co-morbidity	Under 1 (1239)		1-5 years (2252)		6-12 years (1617)		13-17 years (1502)		Total (6610)	
	Number	Deaths	Number	Death	Number	Death	Number	Death	Number	Death
**Cardiovascular diseases**										
Yes	55 (4.4%)	9 (16.4%)	57 (2.5%)	9 (15.8%)	32 (2.0%)	6 (18.7%)	39 (2.6%)	7 (17.9%)	183 (2.8%)	31 (16.9%)
No	1184 (95.6%)	76 (6.4%)	2195 (97.5%)	86 (3.9%)	1585 (98.0%)	70 (4.4%)	1463 (97.4%)	89 (6.0%)	6427 (97.2%)	321 (5.0%)
**Diabetes**										
Yes	20 (1.6%)	6 (30.0%)	32 (1.4%)	4 (12.5%)	27 (1.7%)	4 (14.8%)	28 (1.9%)	2 (7.1%)	107 (1.6%)	16 (14.9%)
No	1219 (98.4%)	79 (6.5%)	2220 (98.6%)	91 (4.1%)	1590 (98.3%)	72 (4.5%)	1474 (98.1%)	94 (6.4%)	6503 (98.4%)	336 (5.2%)
**Chronic pulmonary diseases**^a^										
Yes	5 (0.5%)	1 (20.0%)	27 (1.3%)	5 (18.5%)	18 (1.2%)	0 (0.0%)	16 (1.1%)	5 (31.2%)	66 (1.1%)	11 (16.7%)
No	1006 (99.5%)	63 (6.3%)	2072 (98.7%)	79 (3.8%)	1531 (98.8%)	68 (4.4%)	1403 (98.9%)	82 (5.8%)	6012 (98.9%)	292 (4.9%)
**Liver dysfunction**										
Yes	2 (0.2%)	0 (0.0%)	14 (0.6%)	3 (21.4%)	11 (0.7%)	2 (18.2%)	5 (0.3%)	1 (20.0%)	32 (0.5%)	6 (18.8%)
No	1237 (99.8%)	85 (6.9%)	2238 (99.4%)	92 (4.1%)	1606 (99.3%)	74 (4.6%)	1497 (99.7%)	95 (6.3%)	6578 (99.5%)	346 (5.3%)
**Kidney dysfunction**										
Yes	6 (0.5%)	2 (33.4%)	9 (0.4%)	1 (11.2%)	20 (1.2%)	5 (25.0%)	30 (2.0%)	5 (16.7%)	65 (1.0%)	13 (20.0%)
No	1233 (99.5%)	83 (6.7%)	2243 (99.6%)	94 (4.2%)	1597 (98.8%)	71 (4.4%)	1472 (98.0%)	91 (6.2%)	6545 (99.0%)	339 (5.2%)
**Malignancies**										
Yes	1(< 0.1%)	1 (100.0%)	19 (0.8%)	1 (5.3%)	41 (2.5%)	6 (14.6%)	37 (2.5%)	3 (8.1%)	98 (1.5%)	11 (11.2%)
No	1238 (> 99.9%)	84 (6.8%)	2233 (99.2%)	94 (4.2%)	1576 (97.5%)	70 (4.4%)	1465 (97.5%)	93 (6.3%)	6512 (98.5%)	341 (5.2%)
**Immunodeficiency disorders**										
Yes	2 (0.2%)	0 (0.0%)	9 (0.4%)	0 (0.0%)	15 (0.9%)	1 (6.7%)	13 (0.9%)	2 (15.4%)	39 (0.6%)	3 (7.7%)
No	1237 (99.8%)	85 (6.9%)	2243 (99.6%)	95 (4.2%)	1602 (99.1%)	75 (4.7%)	1489 (99.1%)	94 (6.3%)	6571 (99.4%)	349 (5.3%)

^a^This category included missing values

Among COVID-19 hospitalized children, 14.3% of them had no COVID-19 specific symptom and they just were admitted because of their positive COVID-19 RT-PCR and/or critical condition of their underlying chronic disorder. Fever (41.7%), cough (36%), and dyspnea (27.8%) were the most frequent symptoms in hospitalized children (Table 1).

### Multi variant analysis

#### Age and gender

Gender had no significant correlation with mortality, ICU admission, or ventilator use rate. Infant under 1 year old mortality rate was significantly more than children between 1 and 5 yrs. (*P* value for previously healthy children: 0.001, P value for children with co-morbidity: 0.006) and 6-12 yrs. (P value for previously healthy children: 0.014, P value for children with co-morbidity: 0.029) (Table 3).

**Table 3:** The effect (crude and adjusted ORs) of different characteristics on mortality in Iranian COVID-19 hospitalized children (*N* = 6610)

Characteristics	Crude OR (95%CI)	***P***-value	Adjusted (95%CI) OR	***P***-value
**Demographics**				
**Age**	1.01 (0.99-1.03)	0.166	1.01 (0.99-1.03) ^b^	0.152
**Age < 1 year**	Reference	–	Reference	–
**1-5 years**	0.60 (0.44-0.81)	**0.001**	0.62 (0.44-0.87) ^b^	**0.006**
**6-12 years**	0.67 (0.49-0.92)	**0.014**	0.67 (0.47-0.96) ^b^	**0.029**
**13-17 years**	0.92 (0.68-1.25)	0.623	0.95 (0.68-1.34) ^b^	0.766
**Sex**	1.02 (0.82-1.27)	0.843	1.01 (0.80-1.28) ^b^	0.932
**Comorbidities**				
**Cardiovascular diseases**	3.88 (2.60-5.80)	**< 0.001**	3.27 (2.09-5.11) ^c^	**< 0.001**
**Diabetes**	3.23 (1.88-5.56)	**< 0.001**	2.50 (1.38-4.55) ^c^	**0.003**
**Chronic pulmonary diseases**	3.92 (2.03-7.56)	**< 0.001**	3.21 (1.59-6.47) ^c^	**0.001**
**Liver dysfunction**	4.16 (1.70-10.16)	**0.002**	2.90 (0.96-8.84) ^c^	0.06
**Kidney dysfunction**	4.58 (2.47-8.49)	**< 0.001**	3.42 (1.75-6.67) ^c^	**< 0.001**
**Malignancies**	2.29 (1.21-4.33)	**0.011**	1.88 (0.88-3.98) ^c^	0.101
**Immunodeficiency disorders**	1.49 (0.46-4.85)	0.512	1.06 (0.29-3.89) ^c^	0.925
**Signs and symptoms**				
**No symptoms**	1.36 (1.02-1.79)	**0.033**	1.36 (1.03-1.80) ^a^	**0.030**
**Fever**	0.58 (0.46-0.74)	**< 0.001**	0.59 (0.47-0.75) ^a^	**< 0.001**
**Cough**	1.15 (0.93-1.44)	0.201	1.14 (0.91-1.42) ^a^	0.260
**Headache**	0.39 (0.21-0.71)	**0.002**	0.36 (0.20-0.67) ^a^	**0.001**
**Sore throat**	1.24 (0.87-1.77)	0.238	1.21 (0.84-1.73) ^a^	0.299
**Myalgia**	0.64 (0.43-0.93)	**0.020**	0.60 (0.41-0.88) ^a^	**0.010**
**Dyspnea**	2.66 (2.15-3.31)	**< 0.001**	2.65 (2.13-3.29) ^a^	**< 0.001**
**Diarrhea**	0.24 (0.13-0.43)	**< 0.001**	0.24 (0.13-0.44) ^a^	**< 0.001**

^a^Adjusted for age and sex variables

^b^Adjusted for all comorbidities

^c^Adjusted for other comorbidities, sex and age

#### Underlying co-morbidity

Presence of underlying co-morbidities triples mortality rate (OR: 3.29 [2.36-4.59]). Chronic kidney disease (OR: 3.4 [1.7-6.6]), cardiovascular diseases (OR: 3.2 [2.0-5.1]), chronic pulmonary diseases (OR: 3.2 [1.5-6.4]), and diabetes mellitus (OR: 2.5 [1.3-4.5]), were respectively the most high-risk underlying disease which could increase mortality rate in hospitalized COVID-19 children. Liver disorders (OR: 2.9 [0.9-8.8]), malignancy (OR: 1.8 [0.9-3.9]), and Immune deficiency disorders (OR: 1.06 [0.29-3.89]) did not significantly increase the risk of death in our hospitalized COVID-19 children (Table 3).

#### Sign and symptom

Totally, dyspnea was associated with higher risk of mortality (OR: 2.6, CI 95%: 2.1-3.2, *P* Value: ≺0.001) and patients with no registered COVID-19 specific symptom, were at higher risk of death (OR: 1.3, CI 95%: 1.03-1.8, P Value: 0.03). Fever (OR: 0.5, CI 95%: 0.4-0.7, P Value: ≺0.001), Headache (OR: 0.3, CI 95%: 0.2-0.6, *P* Value: 0.001), myalgia (OR: 0.6, CI 95%: 0.4-0.8, *P* Value: 0.01), and diarrhea (OR: 0.2, CI 95%: 0.1-0.4, P Value: ≺0.001) were significantly associated with reduction in mortality (Table 3).

## Discussion

Among hospitalized Iranian laboratory confirmed cases of COVID-19 (328,541 patients), only 2% were children while near 28% of Iran population are under 18 years old. Mortality rate of Iranian infected hospitalized children was 5.3% and it was 14.8% in children with underlying co-morbidity. The most fatal underlying diseases in Iranian infected children were respectively kidney disorders, cardiovascular disease, chronic pulmonary diseases, and diabetes mellitus. Fever, cough, and dyspnea as typical symptoms of COVID-19 infection are respectively the most frequent symptoms in hospitalized Iranian children and dyspnea was associated with higher (almost three times more) mortality rates.

The center for disease control (CDC) has reported 2 series of data about laboratory confirmed pediatric cases of COVID-19 between February 12 and April 2, and March to July 25, 2020 [24, 25] but they has not mentioned the definite outcomes of children and these papers just mentioned 3 and 1 death, respectively, in their included cases. Also Chinese CDC report 731 pediatric cases and mentioned mild, moderate, severe, and critical ones; Also, they did not mention pediatric symptoms and definite outcomes [26]. Three multicenter reports from China and Italy with more than 100 included cases were reported clinical characteristics of COVID-19 pediatric cases and they mentioned 0 and 1 death in their patients [27–29].

### Age distribution

Among COVID-19 pediatric cases, more frequent (34%) infection that resulted in hospitalization was observed in infants ≺1 year in our study and this age group were at higher risk of mortality in comparison with age groups 1-5 yrs. and 6-12 yrs. Second USA CDC report [25] and the multi centric Italian children study [27] reported 27 and 40% of their hospitalized COVID-19 pediatric cases were infants ≺1 year respectively. Moreover, Chinese CDC reports 30% of ICU admitted COVID-19 pediatric cases were ≺1 year [26]. In addition, it is noteworthy that 54% of critically ill children of this report were ≺1 year. Incomplete vaccination and more sensitivity to dehydration in infants≺1 year may legitimize higher prevalence of COVID-19 complications in this age group. As illustrated in these pediatric studies, young infants are at the highest risk for hospitalization, ICU admission, and death due to COVID-19 infection.

### Signs and symptoms

In Iran, sign or symptom of COVID-19 infection were not registered for about 14% of hospitalized children so it is possible that they were admitted for special care in children with underlying diseases, quarantine, or their symptoms had been missed. Asymptomatic children in Italian multi centric study, a Chinese systematic reviews of case series, and Chinese CDC report were 2.5, 26, and 4.4% in laboratory confirmed cases [26, 27]. In this study hospitalization with no COVID-19 specific symptom, was associated with higher death risk in total inpatient COVID-19 children and this risk was more for children with underlying co-morbidities that had no COVID-19 specific sign or symptoms. Children may be referred with no sign because they were immune deficient and could not show the signs at first. Therefore it is recommended to consider hospital admission of laboratory confirmed COVID-19 cases, especially for children with underlying co-morbidities and infants, even though they have no symptoms.

Fever (41.67%) and cough (36%) as typical symptoms of COVID-19 infection are the most frequent symptoms in hospitalized Iranian children. Also in previous studies, fever and cough have been introduced as the most frequent symptom in COVID-19 infected children and adults [3, 30–32]. The prevalence of fever and cough in our study is compatible with previous studies [24–27].

Dyspnea was the presenting symptom in 27.8% of Iranian hospitalized children and was associated with triplicate risk of mortality in them. Dyspnea was reported much less (9-22%) in other studies [24, 25, 27] and it may be a consequence of a delay in referring to physicians or high prevalence of vitamin D insufficiency (51-62%) in Iranian children [33, 34]. Vitamin D deficiency was also proposed to have association with sever COVID-19 clinical course and infection probability [35–40], Thus, high prevalence of vitamin D deficiency in Iranian children may cause high frequency of more severe cases and consequently, presenting with dyspnea in Iranian hospitalized COVID-19 children.

Fever, myalgia, headache, and diarrhea were associated with death reduction in COVID-19 hospitalized children in our study and it may be due to fast refer of children presenting these symptoms to the hospital. Diarrhea presented in 11.5% of Iranian infected children, while other investigations have pointed that Gastrointestinal (GI) symptom account for 18.4- 42% of COVID-19 symptom in pediatric cases [24, 25, 27]. GI symptoms should be considered as an important possible presenting COVID-19 symptom in children.

### COVID-19 mortality rate in children

Mortality rate of Iranian infected hospitalized children was 5.3% and it triplicate in the children with underlying co-morbidity. Mortality rate in Latin American countries was more than Iran (8.9%) and in China, Italy, and USA were reported much less (≺0.01%).

Chronic disorders identified as the most important risk factor of death in COVID − 19 pediatric cases. We reported mortality rate of PCR positive hospitalized pediatric cases with underlying disorders while this rate was not clarified in previous studies. Good control of this diseases and co-operation of different disciplines in COVID-19 pediatric cases with underlying co-morbidities seems noteworthy and more studies are needed to evaluate aspects of this issue.

Mortality rate in the study of 191 PCR positive hospitalized Latin American children was 8.9% [41]. This study reported higher mortality rate in comparison with Iran. They investigated 14 referral centers in 5 Latin American countries and also included MIS-C cases. These two points interfere with death rate report and may enhance mortality rate in the Latin American study.

We present the largest COVID-19 pediatric cases with definite outcome of acute COVID-19 infection and unfortunately, to the best of our knowledge, Iran has a high registered mortality rate of infected children in comparison with countries with high quality health services. Evaluation of laboratory confirmed pediatric cases in USA, Italy, China have illustrated death, just in children with underlying co-morbidities [25–27]. A large retrospective study in USA have investigated 2430 hospitalized laboratory confirmed COVID-19 pediatric cases and reported 756 severe cases but the exact outcome especially mortality rate was not reported [42]. However, Huge difference in mortality rate of Iranian pediatric cases in comparison with these countries may be a consequence of following reasons; (1) We included all pediatric cases of all COVID-19 active hospital in Iran while other studies were multi centric studies except the USA study which did not report death number; (2) We excluded mild cases that receive outpatient management so our study denominator of mortality rate was smaller; (3) As a consequence of sanctions the quality of health care services was unfavorable in Iran [43].

### Limitations and strengths

#### Our study had some limitations

First, some data were filled in the electronic COVID-19 registry of Iran in an inappropriate manner and we had to use text mining process to repair the primary data and extract our identified variables. However, Iran COVID-19 registry contains these variables; demographic characteristics of children, signs and symptoms during admission, PCR result, ICU admission, ventilation support, admission outcome as discharged or expired and chronic disorders at the time of admission. So, pharmacological treatments and intensive care facilities were not accessed in this study. It is recommended to evaluate children, case by case, for understanding why Iranian children mortality rate is high.

Second, Iran COVID-19 registry data did not include pediatric cases follow up, so MIS cases and long term COVID-19 complication were not evaluated in this study.

Third, we included children ≺18 years old while some studies categorized children as children≺15 years, and this interrupted comparisons. To compensate this contradiction we compared children in different age group according to their biological stage.

Forth, gold standard of COVID-19 infection diagnosis is RT-PCR, but this test’s false negative is 30% in the best conditions of sampling and with the best laboratory processing [44]. Hence, at least two negative results are needed to confirm that child does not have COVID-19 infection. In this study we used Iranian COVID-19 registry in which physicians performed one test in most of the pediatric cases and they manage suspected patients according their laboratory tests (other than R-PCR) or radiologic data. We chose just RT-PCR positive pediatric cases to prevent doubted data.

Despite these limitations, this study is the largest cross sectional study of laboratory confirmed COVID-19 children with definite outcomes.

## Conclusions

COVID-19 mortality in hospitalized Iranian children is relatively high. To tackle these high rates, although hospitalized pediatric cases of COVID-19 are much less than adults, pediatricians should consider children presenting with dyspnea, infants≺ 1 year and children with underlying co-morbidities, as high risk groups for hospitalization, ICU admission, and death.

## Supplementary Information

**Additional file 1: Appendix 1**. Co-morbidities prevalence of each age subgroup in Iranian COVID-19 hospitalized children. **Appendix 2**. Association of clinical symptom with death in Iranian hospitalized children with COVID-19 Infection.

## Data Availability

The data that support the findings of this study are available on request from the corresponding author, Dr. Negar Rezaei.

## Abbreviations

MIS: Multisystem Inflammatory Syndrome
RT-PCR: Reverse Transcription Polymerase Chain Reaction
WHO: World Health Organization
NIMAD: National Institute for Medical Research Development
OR: Odds Ratio
CDC: Center for Disease Control
ACE2: Angiotensin Converting Enzyme 2
GI: Gastrointestinal
