# Supplementary material for: Red flags of poor prognosis in pediatric cases of COVID-19: the first 6610 hospitalized children in Iran
Source: BMC Pediatr. 2021 Dec 10;21:563. doi: 10.1186/s12887-021-03030-2 (PMC8660655; doi:10.1186/s12887-021-03030-2)
Supplement: Supplementary file 1 — Additional file 1: Appendix 1. Co-morbidities prevalence of each age subgroup in Iranian COVID-19 hospitalized children. Appendix 2. Association of clinical symptom with death in Iranian hospitalized children with COVID-19 Infection. [file 12887_2021_3030_MOESM1_ESM.docx]

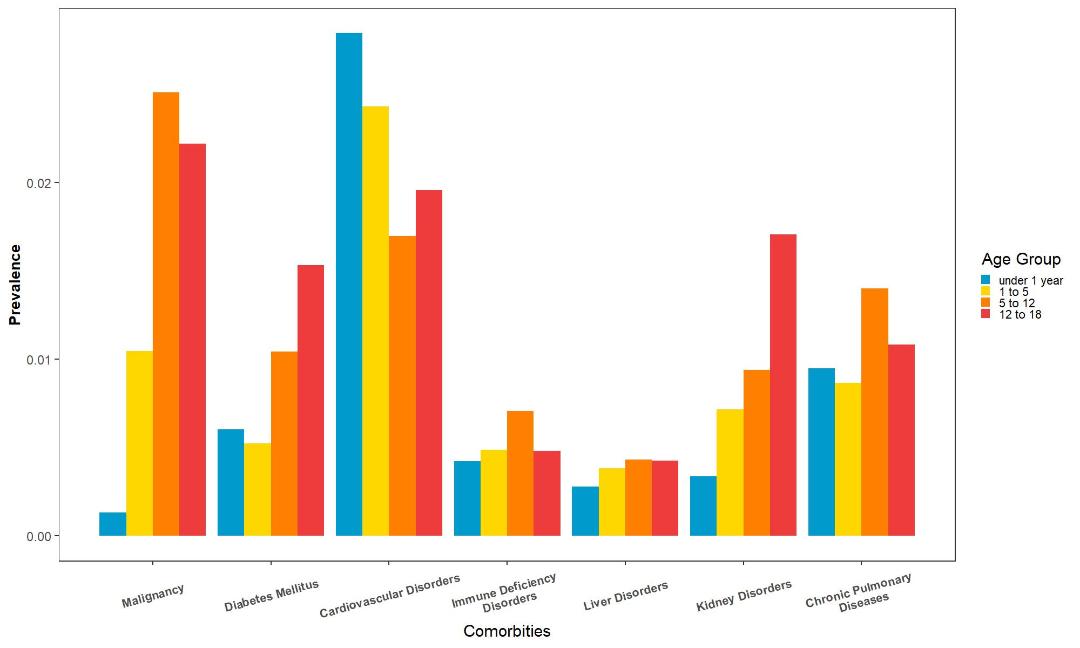


Appendix 1.Co-morbidities prevalence of each age subgroup in Iranian COVID-19 hospitalized children


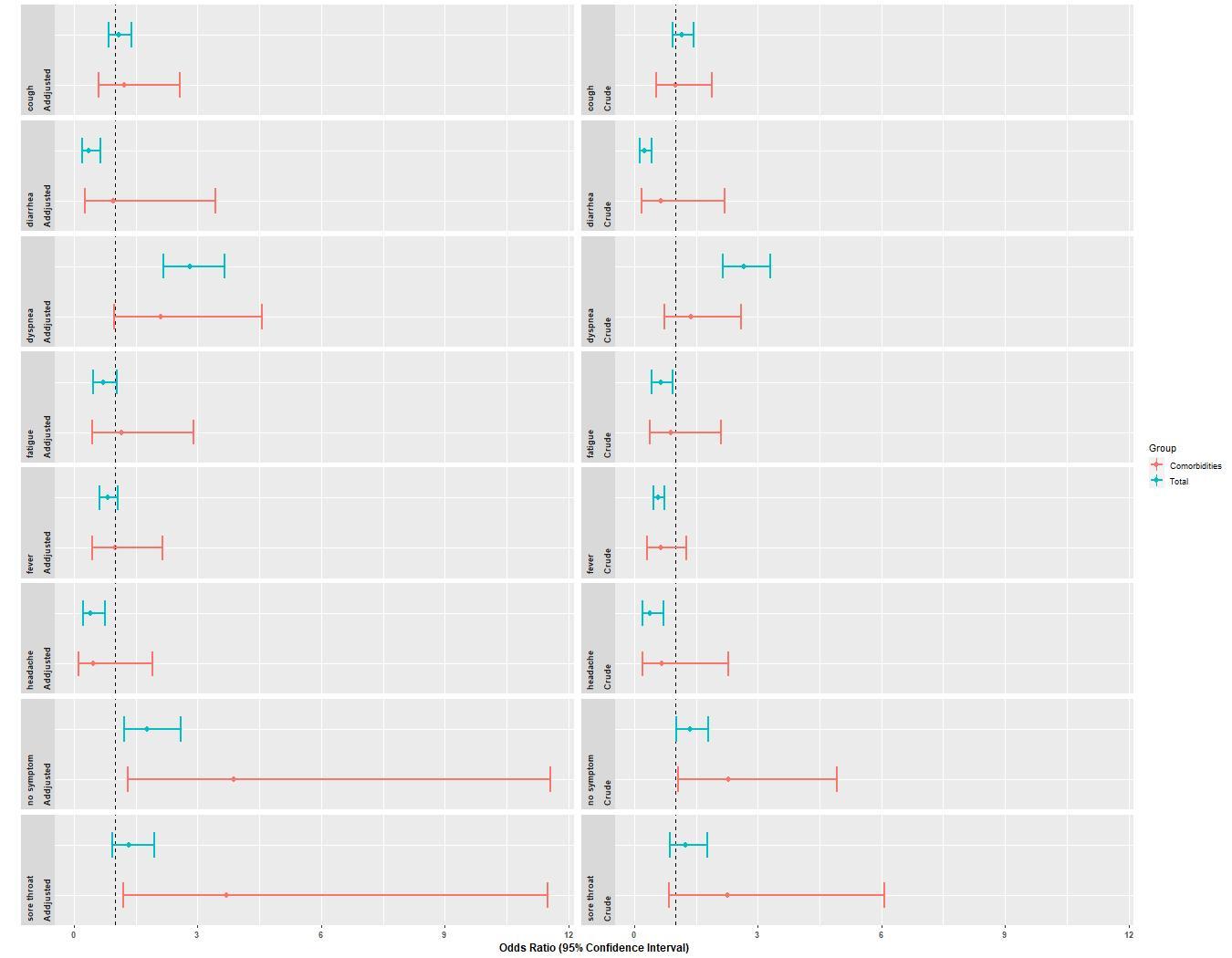


Appendix 2. Association of clinical symptom with death in Iranian hospitalized children with COVID-19 Infection
